# Hair Testing for Classic Drugs of Abuse to Monitor Cocaine Use Disorder in Patients Following Transcranial Magnetic Stimulation Protocol Treatment

**DOI:** 10.3390/biology10050403

**Published:** 2021-05-05

**Authors:** Maria Concetta Rotolo, Roberta Pacifici, Manuela Pellegrini, Stefano Cardullo, Luis J. Gómez Pérez, Diego Cuppone, Luigi Gallimberti, Graziella Madeo

**Affiliations:** 1 National Centre for Addiction and Doping, Istituto Superiore di Sanità, 00161 Rome, Italy; roberta.pacifici@iss.it (R.P.); manuela.pellegrini@iss.it (M.P.); 2 Novella Fronda Foundation, Piazza Castello, 16, 35141 Padua, Italy; stefano.cardullo@gmail.com (S.C.); luigomper@gmail.com (L.J.G.P.); diego.cuppone@yahoo.com (D.C.); luigigallimberti@studiogallimberti.it (L.G.); graziemadeo@gmail.com (G.M.)

**Keywords:** hair testing, cocaine, THC, repetitive transcranial magnetic stimulation (rTMS), biomarker

## Abstract

**Simple Summary:**

Hair testing for classic drugs of abuse offers the possibility of longer detection times (for drug substances) when compared to urine analysis. Hair analysis is routinely used to detect drug abuse in forensic cases and clinical toxicology, whereas it is rarely used at addiction clinics to monitor the clinical efficacy of therapeutic interventions. Here, we explore for the first time whether hair analysis might represent a valid tool to track the clinical improvements in a population of patients with cocaine use disorder (CocUD) who underwent a repetitive transcranial magnetic stimulation (rTMS) treatment.

**Abstract:**

In recent years, hair has become an alternative biological specimen for drug testing in the fields of forensic and clinical toxicology. The advantages of hair testing include larger detection windows (months/years), depending on the length of the hair shaft, compared to those of urine/blood (hours to 2–4 days for most drugs). Segmental hair analysis can disclose a month-to-month (considering 1 cm segment cuts) information of drug exposure (single or repeated) and potentially identify patterns of drug use/administration. Repetitive transcranial magnetic stimulation (rTMS) was recently proposed as a valid tool for therapeutic purposes in addictions, including cocaine use disorder (CocUD). Here, we proposed hair testing analyses of classic drugs of abuse in a clinical setting to monitor the clinical changes in treatment-seeker CocUD patients undergoing protocol treatments with rTMS stimulating the left dorsolateral prefrontal cortex (l-DLPFC). We collected hair samples from nine CocUD patients at different stages from the beginning of treatments. Hair sample analyses revealed significant changes in the patterns of cocaine use, according to the negativity of urine screening tests and the clinical reductions of craving. These data, albeit preliminary, suggest that hair testing analysis of classic drugs of abuse could be extended to clinical settings to monitor the clinical efficacy of innovative therapeutic interventions, such as rTMS.

## 1. Introduction

In contrast to drug testing in conventional matrices, testing of hair may provide data on long-term exposure to drugs. The typical hair sample (3.0 cm cut close to the scalp) identifies drug use as far as three months back, while body hair may go back even further, as opposed to urine analysis, which typically detects drug use from the past 48 to 72 h [1,2,3]. For this reason, hair testing is a well-recognized method for forensic, legal, and clinical purposes, to assess one’s individual history of drugs of abuse [4]. Segmental hair analysis can disclose month-to-month (considering 1 cm segment cuts) eventual repeated chronic exposures and, in some instances, identify patterns of drug use/administration [5]. Moreover, hair drug testing can be used in epidemiological and clinical studies to objectively assess repetitive exposure to a certain drug in specific patient populations [6]. Recently, repetitive transcranial magnetic stimulation (rTMS) was proposed as a valid tool for therapeutic purposes in addictions, including cocaine use disorder (CocUD) [7,8]. Indeed, the traditional strategies to attenuate drug effects (e.g., with medications or psychological interventions) have not resulted in effective therapeutic interventions for cocaine addiction. Here, we test whether hair testing for drugs of abuse is a useful methodology in a clinical setting to monitor the clinical changes observed in CocUD patients who underwent the rTMS protocol treatment stimulating the left-DLPFC [9]. In this study, we used a validated analytical methodology, already applied in several clinical and epidemiological studies [10,11], to assess the pattern changes of drug use in patients who underwent innovative protocol treatments with rTMS.

## 2. Materials and Methods

### 2.1. Chemicals and Reagents

Cocaine (COC), benzoylecgonine (BEG), morphine (MOR), 6-monoacetylmorphine (6-MAM), codeine (COD), methadone (MTD), 2-ethylidene-1,5-dimethyl-3,3-diphenylpyrrolidine (EDDP), amphetamine (A), methamphetamine (MA), methylenedioxyamphetamine (MDA) 3,4-Methylenedioxymethamphetamine (MDMA) Delta-9-tetrahydrocannabinol (THC), 11-nor-Delta 9 tetrahydrocannabinol-9-carboxylic acid (THC-COOH), and respective deuterated standards used as internal standards (ISs) were supplied by LGC Standards (Sesto San Giovanni Italy). N-O-Bis(trimethylsilyl)trifluoroacetamide/trimethylchlorosilane (BSTFA with 1% TMS) and N-methyl-N-(trimethylsilyl)trifluoroacetamide (MBTFA) were obtained from Sigma-Aldrich (Milano, Italy). All reagents of analytical grade, such as methanol, dichloromethane *tert*-butyl methyl ether, were obtained from Carlo Erba (Milano, Italy).

### 2.2. Study Design

This research study focused on hair testing analyses of classic drugs of abuse in nine treatment-seeker subjects diagnosed with CocUD, according to the Diagnostic and Statistical Manual of Mental Disorders 5th Edition (DSM–5) [12], as assessed by a clinical psychiatrist specialized in substance use disorders (SUDs) [9]. Patients were admitted to a specialized clinical center for addiction treatment in Padua, Italy, from 2013 to 2020, and they voluntarily underwent a protocol treatment with rTMS stimulating the left dorsolateral prefrontal cortex (l-DLPFC). Participants had at least 3 cm of scalp hair and provided a written informed consent authorizing the administration of the rTMS protocol and the use of their data for research purposes. They were 20 to 56 years old, and met diagnostic criteria for CocUD according to the DSM–5. Participants were also informed that the data collected would be managed according to the law on privacy and the Legislative Decree No. 196 of 30 June, 2003, “Personal Data Protection Code”, ensuring anonymity. The current study is part of a larger study approved by the Ethical Committee at Padua Teaching Hospital (protocol number: 4743/U6/19). Exclusion criteria included a lifetime history of other psychiatric diseases, including schizophrenia, bipolar disorder, other psychoses, or unstable medical illnesses. During the clinical management, cocaine use was assessed by urine screening, self-reports, and reports by collateral informants. rTMS treatment was administered to each patient by a trained clinical physiologist using a medical device (MagPro R30) targeting the l-DLPFC. Treatment consisted of twice-daily rTMS sessions for the first five consecutive days of treatment. Subsequently, rTMS was administered at weekly intervals (twice-daily sessions) for 11 consecutive weeks. The number of weekly sessions was adjusted according to the specific needs and the clinical response of each patient. Resting motor threshold (rMT) and stimulation parameters were defined as previously reported [8]. Hair drug testing focused on assessing the concordance between hair testing and the clinical assessment of drug use (i.e., urine screening at time of enrollment, self-report, and collateral informants’ reports). Therefore the main objectives for hair drug testing were: (a) to examine the correspondence between self-reported protracted abstinence from cocaine use and hair analysis in CocUD patients after at least 1 year from rTMS treatment start point; (b) to evaluate the correspondence between self-reported cocaine consumption frequency level and hair drug testing at clinical intake; and (c) to investigate the level of accordance between the clinical assessment of drug use and the hair analysis before and after rTMS treatment. Hair samples were collected using laboratory-recommended procedures from nine patients with CocUD. Hair length ranged from 4 cm to 40 cm. Hair length was of at least 3 cm; all strands were examined as unique segments. For hair samples of 6 cm length, two segments (proximal and distal) were analyzed, representing the two last life trimesters [2,3,12]. No hair from other body parts were considered, as it was demonstrated that only head hair, growing on average of 1 cm per month, can account for timely past exposure to a drug [2,3].

### 2.3. Preparation of Calibration Standards and Quality Control Samples

Stock standard solutions containing all substances at 1 mg/mL concentrations were prepared in methanol. Working solutions at concentrations 1 μg/mL were immediately prepared by dilution of the stock standards with methanol and stored at −20°C until analysis. The internal standard (IS) working solutions were used at a concentration of 1 μg/mL. The method linearity for each compound was investigated in the range of 0.1–25 ng/mg. Calibration curves were obtained with five calibration levels by adding 0.1, 0.5, 1, 5, 10, and 25 ng analytes under investigation per mg hair. The solutions were prepared daily for each analytical batch by adding suitable amounts of working solutions to 25 mg of a pre-checked drug-free hair pool, donated by laboratory personnel. Quality control (QC) samples of 0.3, 4, and 20 ng analytes under investigation per mg of hair were prepared daily in drug-free hair samples, and used for calculation of validation parameters. Deuterated internal standards (10 ng each) were added, and samples were analyzed following the complete procedure. Dilution integrity was tested for over-the-curve samples with a concentration 5 and 10 times higher than the highest calibrators, with a dilution in methanol before sample treatment, verifying precision and accuracy to be within 15%.

### 2.4. Sample Preparation for Analysis of COC, BZE, MOR, 6-MAM, COD, MTD, and EDDP in Hair Samples

Hair samples were cut close to the scalp in the vertex region. Hair strands were then divided in two different segments depending on the length of the hairs. The first was cut at 3 cm from the proximal region, representing hair growth in the last three months, and the second at 3 cm to account approximately for the last 6 months. These cuts were decided according to international literature on the subject [13], and considering the mean length of hair samples from the consumers. Classic drugs of abuse and principal metabolites, such as COC, BZE, MOR, 6-MAM, COD, MTD, and EDDP were analyzed in hair samples by gas chromatography–mass spectrometry (GC/MS). Drug-free hair samples were decontaminated with 3 mL dichloromethane washings; after drying at room temperature, 25 mg of hair was cut into small pieces (about 2–3 mm). Hair samples were fortified with ISs, and incubated with phosphate buffer 0.1 M pH 6 overnight at 45 °C. The samples, after cooling and centrifugation, were extracted with solid-phase extraction by Bond Elut Certify columns, following the instructions of the manufacturer. After elution and evaporation of the organic layer, the analytes were derivatized with 50 µl BSTFA with 1% TMS at 70 °Cfor 30 min, and the derivatives were injected to GC/MS.

### 2.5. Sample Preparation for Analysis A, MA, MDA, MDMA in Hair Samples

The pooled drug-free hair and hair samples (25 mg) were washed with 3 mL dichloromethane and allowed to dry at room temperature. One milliliter of 1 M sodium hydroxide added with ISs was added to every hair sample. The samples were then extracted with 3 mL of *tert*-butyl methyl ether, and shaken with a vortex for 2 min. The organic layer was evaporated to dryness under nitrogen flux at a temperature lower than 40 °C. Trifluoroacetyl derivatives were formed by reaction with 50 µL of MBTFA as a derivatization agent, in a dry bath, at 70 °C for 30 min.

### 2.6. Sample Preparation for Analysis of THC and THC-COOH

Hair samples (25 mg) fortified with deuterated standards were incubated in a sodium hydroxide solution for 20 min at 95 °C and extracted with 3 mL of n-hexane: ethyl acetate (9:1). The organic extract was evaporated under a nitrogen stream at 40 °C. For extraction of THC-COOH metabolite, the remaining aqueous sample was acidified with 1 mL of 1 N HCl (resulting pH 4–5), extracted with 3 mL n-hexane: ethyl acetate (9:1) and centrifuged again to obtain a second extract. The combined organic phases were evaporated to dryness under nitrogen at 40 °C, and were then derivatized with 50 µL of 99:1 (*v*/*v*) BSTFA/TMCS at 70 °C for 30 min. Finally, 1 μL of the sample was injected into GC/MS.

### 2.7. GC–MS Instrumentation

The GC–MS instrument consisted of a gas chromatographer Agilent 7890 A coupled with 5975 C MSD (Agilent Technologies, Palo Alto, CA, USA). Ultra Inert GC column Zebron (ZB-Drug-1, 15m × 250 µm i.d., film thickness 0.25 µm; Phenomenex, Milan, Italy) was installed. Analytes separation for all drugs of abuse was achieved on a fused silica capillary column (HP-5MS, 30 m × 0.25 mm i.d., film thickness 0.25 m) (Agilent Technologies, Milan, Italy). The oven temperature for classic drugs of abuse was programmed at 80 °C for 1 min, increased to 230 °C at 35 °C/min, and then raised to 290 °C at 10 °C/min, and held for 10 min. To analyze amphetamine, the oven temperature was initially maintained at 70 °C during 2 min and programmed to 200 °C at 40 °C per min, and subsequently to 290 °C at 10 °C per min. Helium was used as a carrier gas at a flow rate of 1.2 mL/min. For cannabinoids determination, the oven temperature was programmed at 140 °C for 2 min, increased to 290 °C at 20 °C/min, and held for 10 min. For all substances, split injection mode (15:1) was used. Helium (purity 99%), with a flow rate of 1 mL/min was used as a carrier gas. The injection port, ion source, quadrupole, and interface temperatures were 260 °C, 230 °C, 150 °C, and 280 °C, respectively. The dwell time was 0.2 msec. The electron-impact (EI) mass spectra of analytes were recorded by the total ion-monitoring mode (scan range 40–550 *m/z*) to determine retention times and characteristic mass fragments. Retention times and qualifying ions of all drugs monitored in the selected-ion-monitoring (SIM) mode are reported in Table 1. The ion ratio acceptance criterion was a deviation of ≤20% of the average of ion ratios of all the calibrators. The underlined ions were used for quantification. As hair is a complex matrix with numerous interferences, which can interfere with the instrument signals, ions with the highest masses were chosen for quantification, although they were not the most abundant. This was specifically made to eliminate interference and have certain and safe results without affecting the sensitivity of the method.

### 2.8. Validation of Analytical Method

Prior to application to real samples, the method was tested in a 3-day validation protocol following the accepted criteria for bioanalytical method validation [13,14]. Selectivity, matrix effect, linearity, limits of detection (LOD), and quantification (LOQ), precision, accuracy, and recovery were assessed. Absolute analytical recoveries were calculated by comparing the peak areas obtained when QC samples were analyzed by adding the analytical reference standards in five different drug-free hair samples, prior (and after) the extraction procedure (five replicates at each concentration). Matrix effects were determined by comparing the peak area of six different blank samples fortified with standards at QC concentrations after the extraction procedures were compared to the peak areas of pure diluted substances. Validation parameters were calculated using five different daily replicates of QC samples (low, medium, and high QCs) along three subsequent working days. Linearity was determined by least-square regression with 1/×2 weighting. Acceptable linearity was achieved when the coefficient of determination was at least 0.99 and the calibrators were quantified within ±20% at the LOQ and ±15% at other concentrations. The LOD and LOQ were evaluated with decreasing analyte concentrations in the hair samples. LOD was defined as the lowest analyte concentration that could be detected and identified with a given degree of certainty. Standard deviation (SD) of the mean signal-to-noise level over the retention time window of each analyte was used to determine LOD. A minimum requirement for signal-to-noise of 3 was widely accepted. LOQ was the lowest concentration that met LOD criteria and a signal-to-noise ratio of at least 1. Precision and accuracy were expressed as the coefficient of variation (CV%) and percentage of error (E%) of the measured values, respectively, were expected to be less than 20%.

## 3. Results

### 3.1. Validation Results

The retention times of all substances are reported in Table 1. Drug-free hair samples were injected after the highest point of the calibration curve did not present any traces of carryover. Washing solvents, analyzed for any eventual external contamination, were free of drugs and respective metabolites. Calibration results, limits of detection (LOD), and limits of quantification (LOQ) for analytes under investigation are reported in Table 2.

The intra-and inter-assay precision and accuracy data are presented in Table 3; values were always lower than 15%.

No additional peaks due to endogenous substances, which could have interfered with the detection of the analytes under investigation, were observed in drug free hair samples. No psychoactive drugs, such as benzodiazepines (diazepam, alprazolam, flunitrazepam, and oxazepam) or major antidepressants (e.g., sertraline, fluoxetine, escitalopram, paroxetine) interfered with the assay. Blank hair samples injected after the highest point of the calibration curve did not present any traces of carryover. Absolute analytical recovery (mean ± SD of 15 replicates) ranged from 90.5% ± 4.2 for 6-MAM, 94.3% ± 2.6 for MOR, 88.9% ± 3.2 for COD, 90.1% ± 5.2 for COC, 91.4% ± 2.8 for BZE, 86.2 ± 4.5 for MTD, 88.2% ± 5.3 for EDDP, 92.2% ± 3.2 for A, 90.1% ± 3.2 for MA, 91.4% ± 2.1 for MDMA, 90.3% ± 3.2 for MDA, 80.6 ± 6.2% for THC, and 81.7 ± 4.3% for THC-COOH. The matrix effect showed less than 10% analytical suppression due to endogenous substances for both the analytes

### 3.2. Sample Results

As shown in Table 4, the method was successfully applied to real hair samples of CocUD patients undergoing the rTMS protocol treatment. Nine subjects were enrolled in this pilot study to assess the usefulness of drug hair testing for monitoring the clinical efficacy of an innovative therapeutic intervention, such us rTMS, for cocaine addiction.

In Table 4, results from GC/MS analysis are shown. The hair sample from case 1 was collected at 77 days from the beginning of treatment. Despite the patient reporting no cocaine use in the last 30 days before starting treatment, and all urine screening tests being negative, hair analysis showed a medium positivity (hair cocaine concentration between 4 and 20 ng/mg [15]) in the distal hair segment (3–6 cm) and a low positivity in the proximal hair segment (0–3 cm). Hair analysis of case 2, sampled at 219 days from the beginning of treatment, demonstrated a reduction of cocaine use from medium to low positivity, consistent with the urine screening tests performed at each clinical follow-up. According to self-reported consumption and urine analysis, the case 3 hair analysis demonstrated a high positivity level of cocaine use. The hair from case 4 was collected at day 1 of the treatment, showing a high positivity level for cocaine consumption. Unfortunately, it was not possible to collect further samples as the patient voluntarily interrupted the treatment. In cases 6 and 7, hair analyses confirmed abstinence, as reported by the patients, and shown by the regular urine analyses. However, hair analyses revealed that cases 6 and 7 used cannabis during the treatment, as demonstrated by the positivity of THC and its metabolite THCCOOH (shown in Table 5). Hair analyses confirmed the suspension of cocaine use in both cases 8 and 9, which were sampled at 142 and 164 days from the beginning of the treatments, respectively. Accordingly, self-reported consumption and urine screening tests were negative for cocaine use during the rTMS treatment.

## 4. Discussion

Drug testing in hair is important in clinical and forensic settings, to prove consumption, and help evaluate one’s level of impairment. Hair testing is particularly useful in drug rehabilitation programs, workplace drug testing, and driver’s license renewals. This stable matrix may be used to confirm long-term exposure to drugs over a period of weeks to years, depending on the length of hair collected. To our knowledge, this study proposes, for the first time, the use of a well-established methodology for drug testing in hair in a clinical setting, to monitor the therapeutic effects of an innovative treatment for cocaine addiction. rTMS stimulating l-DLPFC is receiving increasing attention as a potential treatment for addictions, including CocUD [8,9]. Indeed, the traditional strategy with medications or psychological interventions to attenuate drug effects have not resulted in effective therapeutic interventions for cocaine addiction. Several clinical findings suggest that rTMS of l-DLPFC may represent a human translation of preclinical findings on rodents—that cocaine seeking is attenuated by optogenetic activation of specific prefrontal circuits [8]. Drug consumption is routinely assessed by patients’ self-reports, reports from patient referrals, and urine screening tests. However, these methods might be biased by several factors, and urine-screening analysis offers a narrow time window to verify the positivity to drugs. Therefore, we investigated whether hair drug testing could be used to ascertain the concordance between the clinical assessment of drug consumption and the hair analysis. In this pilot study, we collected hair samples from nine CocUD patients, at different stages, from the beginning of rTMS treatments. In four CocUD patients, we were able to confirm the protracted abstinence, as self-reports, urine screening tests, and hair analyses were negative for cocaine and its metabolite. Likewise, hair analysis results showed a significant reduction of cocaine use in one patient, in accordance with the self-report and urine screening test. When evaluating the correspondence between self-reported cocaine consumption frequency levels and hair drug testing at clinical admission—hair drug testing in two CocUD patients showed a degree of consumption, in agreement with the patients’ self-reports. Moreover, hair drug testing helped us investigate whether CocUD patients were substituting cocaine consumption with other drugs of abuse. Interestingly, despite a significant clinical improvement in cocaine use, two patients started using cannabis, as demonstrated by the hair result positivity for THC and its metabolite. We are aware that this study involves a small number of patients, but these preliminary data provide novel insights on using a well-established hair drug testing methodology to rigorously monitor the clinical changes of drug patterns of use in patients undergoing innovative treatments, such as rTMS.

## 5. Conclusions

The LC–MS/MS method developed for the simultaneous determination of The GC–MS method, developed for the simultaneous determination of 13 drugs of abuse in hair samples, is simple, rapid, and robust. The method was applied to real hair samples collected from CocUD patients undergoing innovative addiction treatments. Despite our results coming from a very small number of patients, we suggest that this methodology be considered as a support measure, to evaluate the decrease in cocaine consumption and changes in the patterns of drug use.

## Figures and Tables

**Table 1:** Retention time (Rt), monitored ions (*m/z*) for analytes under investigation and deuterated internal standards.

Compounds	GC/MS		
	Rt	*m/z* ions	*m/z* ions deuterated IS
6 _MAM-TMS	7.9	287,340,399	290,343,402
MOR-TMS	7.0	401,414,429	404,417,432
COD-TMS	6.7	196,234,371	316,346,374
COC	6,0	82,182,303	85,185,306
BZE-TMS	6,2	82,240,361	85,243,364
MTD	5,2	72,223,294	81,233,303
EDDP	4,6	220,262,277	223,265,280
AP-TFA	4.8	91,118,140	97,124,146
MA-TFA	5.0	91,118,154	96,123,159
MDMA-TFA	5.8	135,154,162	140,159,167
MDA-TFA	6.0	135,162,275	140,167,280
THC-TMS	11.5	303,371,386	306,374,389
THC-COOH-TMS	9.5	371,473,488	374,476,491

Underlined *m/z* ions were used for quantification.

**Table 2:** Calibration parameters, limits of detection (LOD), and limits of quantification (LOQ) for analytes under investigation.

Compounds	Slope; mean ± SD ^a^	Intercept mean ± SD ^a^	Correlation Coefficient (r^2^) mean ± SD ^a^	LOD ng/mg	LOQ ng/mg
6 _MAM-TMS	0.010 ± 0.021	0.195 ± 0.003	0.998 ± 0.002	0.05	0.1
MOR-TMS	0.021 ± 0.032	0.064 ± 0.001	0.998 ± 0.003	0.05	0.1
COD-TMS	0,0064 ± 0.011	0.021 ± 0.001	0.997 ± 0.002	0.05	0.1
COC	0.0071 ± 0.011	0.083 ± 0.005	0.991 ± 0.001	0.05	0.1
BZE-TMS	0.0045 ± 0.042	0.091 ± 0.002	0.996 ± 0.002	0.05	0.1
MTD	0.0058 ± 0.010	0.064 ± 0.001	0.998 ± 0.002	0.05	0.1
EDDP	0.186 ± 0.021	0.016 ± 0.002	0.994 ± 0.004	0.05	0.1
AP-TFA	0.022 ± 0.001	0.413 ± 0.004	0.990 ± 0.004	0.05	0.1
MA-TFA	0.037 ± 0.002	1.141 ± 0.001	0.993 ± 0.002	0.05	0.1
MDMA-TFA	0.037 ± 0.002	2.323 ± 0.001	0.998 ± 0.001	0.05	0.1
MDA-TFA	0.0044 ± 0.011	0.434 ± 0.002	0.991 ± 0.005	0.05	0.1
THC-TMS	0.011 ± 0.010	0.021 ± 0.005	0.999 ± 0.004	0.03	0.1
THC-COOH-TMS	0.062 ± 0.021	0.028 ± 0.001	0.998 ± 0.001	**0.02**	**0.05**

^a^ Mean ± SD (standard deviation) of 5 replicates.

**Table 3:** Intra-day (*n* = 5) and inter-day (*n* = 15) imprecision and inaccuracy for analytes under investigation.

Compounds	Intra-Day Imprecision (CV%) in Quality Control Hair Samples			Intra-Day Inaccuracy (Error%) in Quality Control Hair Samples			Inter-Day Imprecision (CV%) in Quality Control Hair Samples			Inter-Day Inaccuracy (Error%) in Quality Control Hair Samples		
	Low QC	Medium QC	High QC	Low QC	Medium QC	High QC	Low QC	Medium QC	High QC	Low QC	Medium QC	High QC
6-MAM-TMS	3.4	6.8	5.3	5.8	7.2	4.3	3.2	4.3	5.7	3.8	4.7	7.3
MOR-TMS	5.1	4.6	8.1	2.8	5.5	6.8	4.3	5.7	6.1	2.4	5.8	3.4
COD-TMS	5.5	3.8	4.5	6.6	2.8	4.8	5.9	4.1	3.3	6.8	9.1	4.2
COC	3.1	4.2	5.8	5.5	4.3	6.6	4.7	3.2	2.8	5.9	6.6	6.1
BZE-TMS	5.8	2.3	4.1	3.3	5.2	4.1	6.6	5.4	7.2	8.0	5.6	6.6
MTD	4.4	3.2	5.8	2.5	7.2	4.1	6.7	8.2	2.1	4.9	5.2	7.1
EDDP	2.2	5.1	4.3	4.8	2.4	6.6	5.9	2.5	4.3	9.1	5.7	8.3
AP-TFA	6.8	3.4	5.3	6.1	6.5	7.4	8.1	4.3	2.8	4.7	5.2	2.8
MA-TFA	5.1	4.5	8.1	6.6	4.8	6.4	7.2	4.2	7.2	6.8	5.5	6.7
MDMA-TFA	3.3	6.3	4.2	8.1	7.1	5.5	4.6	8.6	5.3	7.3	8.5	2.5
MDA-TFA	4.2	2.5	5.3	6.6	8.2	4.2	5.3	4.9	8.1	2.4	5.2	6.3
THC-TMS	5.3	4.2	2.6	4.4	3.2	7.2	5.3	4.8	5.1	6.6	8.1	4.3
THC-COOH-TMS	2.2	3.2	6.3	5.4	8.2	4.6	6.2	5.1	7.1	5.3	4.3	2.1

**Table 4:** Cocaine urine screen and pre–post treatment hair analyses outcomes in CocUD patients undergoing rTMS treatment.

Patient	Days in treatment at sampling	Last month cocaine frequency level at baseline ^1^	Days from last use reported	Proportion of in treatment cocaine-positive urine screen (Pos/Tot)	COC (Dis/Prox) (ng/mg)	BEG (Dis/Prox) (ng/mg)	Level of consumption based on hair analysis at baseline ^2^	Level of consumption based on hair analysis at sampling ^2^
Case 1	77	Abstinence	158	0/10	4.10/3.10	0.60/0.40	Medium positivity	Low positivity
Case 2	219	Low frequency	176	7/25	4.50/3.60	1.90/1.60	Medium positivity	Low positivity
Case 3	39	High frequency	0	2/6	85.10/46.30	20.70/10.70	High positivity	High positivity
Case 4	1	Low frequency	1	1/1	58.50 *	12.90 *	High positivity *	High positivity *
Case 5	92	Low frequency	99	0/14	16.70/0.50	0.60/0.40	Medium positivity	Low positivity
Case 6	210	High frequency	280	0/27	1.20/Neg	2.10/Neg	Low positivity	Absence
Case 7	166	Abstinence	413	0/10	0.70/Neg	0.50/Neg	Absence	Absence
Case 8	142	Abstinence	232	0/16	1.10/Neg	2.20/Neg	Low positivity	Absence
Case 9	164	High frequency	165	0/14	2.07/Neg	1.06/Neg	Low positivity	Absence

CocUD: cocaine use disorder; rTMS: repetitive transcranial magnetic stimulation; Pos: number of urine assay positive for cocaine determined by qualitative detection methods; Tot: total number of urine assay for qualitative detection of cocaine; Dis: distal segment of hair specimen (3–6 cm); Prox: proximal segment of hair specimen (0–3 cm); COC: cocaine; BEG: benzoylecgonine; N.D.: not determined; Neg: negative. ^1^ Last month cocaine frequency level was categorized according to the clinical validation from Roos et al. [15] as follows: abstinence (0 days/month), low frequency (1–4 days/month), and high frequency (5+ days/month). ^2^ Level of cocaine consumption based on hair analysis were established according the proposed interpretation of Pepin and Gaillard (1997) [16] as follow: absence (hair cocaine concentration lower than 1 ng/mg), low positivity (hair cocaine concentration lower than 4 ng/mg), medium positivity (hair cocaine concentration between 4 and 20 ng/mg) and high positivity (hair cocaine concentration higher than 20 ng/mg). * Levels of consumption based on hair analysis at treatment baseline and at sampling time match; therefore, in this case, we consider only the COC/BEG values of the proximal hair specimen segment.

**Table 5:** THC and THCCOOH hair analyses results from patients 6 and 7.

Patient	THC (Dis/Prox) (ng/mg)	THCCOOH (Dis/Prox) (ng/mg)
Case 6	Neg/2.8	Neg/0.002
Case 7	Neg/0.2	Neg/0.002

## Data Availability

All data generated or analyzed during this study are included in this published article.
